# The zero effect: voxel-based lesion symptom mapping of number transcoding errors following stroke

**DOI:** 10.1038/s41598-017-08728-x

**Published:** 2017-08-23

**Authors:** Marleen Haupt, Céline R. Gillebert, Nele Demeyere

**Affiliations:** 1 General and Experimental Psychology, Department of Psychology, Ludwig-Maximilians-Universität München, Munich, Germany; 2 Department of Experimental Psychology, University of Oxford, Oxford, UK; 3 Laboratory of Experimental Psychology, Department of Brain & Cognition, University of Leuven, Leuven, Belgium

## Abstract

Zero represents a special case in our numerical system because it is not represented on a semantic level. Former research has shown that this can lead to specific impairments when transcoding numerals from dictation to written digits. Even though, number processing is considered to be dominated by the left hemisphere, studies have indicated that both left as well as right hemispheric stroke patients commit errors when transcoding numerals including zeros. Here, for the first time, a large sample of subacute stroke patients (N = 667) was assessed without being preselected based on the location of their lesion, or a specific impairment in transcoding zero. The results show that specific errors in transcoding zeros were common (prevalence = 14.2%) and a voxel-based lesion symptom mapping analysis (n = 153) revealed these to be related to lesions in and around the right putamen. In line with former research, the present study argues that the widespread brain network for number processing also includes subcortical regions, like the putamen with connections to the insular cortex. These play a crucial role in auditory perception as well as attention. If these areas are lesioned, number processing tasks with higher attentional and working memory loads, like transcoding zeros, can be impaired.

## Introduction

The concept of zero is considered to be a great achievement of humankind. Even though positive numbers still represent an abstract concept, they correspond to and represent entities in real life. Zero, however, is not used in operations of daily life which include counting or enumerating. To understand the concept of zero we have to move beyond empirical experience and form an abstract, mental category. Even though no elements are present in this category, this absence in itself also forms a mathematical object^1^. The aim of the present study is to address the question how this complex concept of zero is represented in the human brain during number transcoding.

Several theoretical models have attempted to explain the process of number transcoding which is defined as the transformation of an Arabic number from a given format (e.g. phonological, hearing the word forty) into a required, other format (e.g. writing as numerals, 40). In the semantic-abstract model the verbal entry form is translated into a base-10 semantic representation by the system’s comprehension mechanism^2^. The abstract semantic representation subsequently activates the appropriate lexical and syntactic production system. This is realised by planning a frame containing the appropriate number of slots in which the digits, corresponding to the basic values, can be inserted^2^. According to the lexical-semantic model^3, 4^, the corresponding semantic representation is indeed abstract, but does, beyond that, also have an internal structure which is tied to the respective verbal code. Based on the semantic expression, the production of the numerals follows the concatenation (product relationship, e.g. 3 × 100 = 300 with the original number 3 just being concatenated with the “00” being indicative for the construct of “hundred”) rule and overwriting (sum relationships, e.g. 1000 + 24 = 1024 with 24 overwriting the last two digit slots of 1000) rule. The overwriting rule is learned later in life and therefore considered to be more complex^3^.

Within the transcoding of complex numerals, the production of the numeral zero represents a special case because it is not represented on the semantic level^5^. Therefore, zeros would leave empty slots in the frame underlying the semantic-abstract model even though they would have to be retrieved at the lexical production level. In contrast, different mechanisms in the lexical-semantic model could lead to the production of zeros. They could either be derived from semantics or syntactically produced. Lexical zeros, e.g. the zeros in 10 or 90, are a numerical concept which does not involve any kind of production rule. Instead, they are represented within lexical primitives which lead to a direct production of the zero with the preceding digit building a merged Arabic form. On the other hand, syntactic zeros do not build a unit with the preceding digit but result from a concatenation operation with (e.g. 26007) or without (e.g. 26000) an added overwriting operation^5^. Hence, children who have not mastered the overwriting rule in their development yet, mainly have problems transcoding numbers with zeros because they are the only ones requiring that rule.

This is not solely a developmental phenomenon, it can also be seen in neuropsychological patient populations. In line with the lexical-semantic model, a patient with left cerebrovascular damage was found to have difficulties writing complex numerals containing syntactic zeros (e.g. 807) while no difficulties emerged from numerals with lexical zeros (e.g. 80)^5^. This finding confirms former neuropsychological studies reporting errors in transcoding zeros in small samples consisting of only left hemispheric patients^6, 7^. These studies are in accordance with a long line of number processing research stating that the left hemisphere, especially the left parietal lobe, plays an important role in number processing^8–12^. In a PET study, cerebral networks involved in processing numbers were localized^13^. Consistent with former neuropsychological literature, the authors found a significant activation of the left parietal lobes and left precentral gyrus for two processing levels. In the first instance, these areas showed a general activation due to working memory and attentional processes which was not restricted to number processing but also occurred for non-numerical stimuli like symbols. In addition, the parietal areas were found to be significantly involved in processing the magnitude of numerical information.

Although the left hemisphere seems to dominate, right hemispheric function should not be neglected. When contrasting numerical comparison, multiplication and subtraction tasks, Chochon, Cohen, van de Moortele, and Dehaene differentiated between three different patterns in a functional MRI study^14^. A stronger right hemispheric intraparietal and frontal activation was found for the comparison task, while the left hemispheric activation predominated in the multiplication task. For the subtraction task, a bilateral activation pattern was found. Dehaene *et al*. later proposed three parietal circuits for number processing^12^.

However, the above reported imaging studies tested and compared Arabic digit processing, non-symbolic and symbolic magnitude comparison and different types of calculations. None specifically investigated transcoding of zeros. Furumoto tested eighteen patients with right cerebral infarctions and demonstrated frequent errors in transcoding numerals which include zeros (e.g. “2306” → “23006”) proposing that the errors of patients with right cerebral damage can be purely explained by misallocations of zeros^15^. These results are in line with other studies reporting transcoding errors in patients with right hemispheric damage^16–19^. Nevertheless, these comparable results were not succeeded by consistently drawn conclusions. Patients’ transcoding errors were explained by impairments of different cognitive functions such as cognitive processing^19^ or spatial abilities^17^. Furumoto proposed that pure misallocations of zeros are a unique phenomenon independent from other cognitive dysfunctions^15^.

To explicitly address the necessary contributions of the right cerebral hemisphere to number transcoding processes, a recent study by Benavides-Varela *et al*. assessed 22 right-brain-damaged patients with two number transcoding tasks: reading Arabic numerals and writing to dictation in the Arabic code^20^. Voxel-based lesion symptom mapping (VLSM) was used to identify brain regions that were significantly associated with committing zero errors in transcoding. The results of their behavioural analysis confirmed that patients with right hemispheric lesions commit significantly more zero errors than healthy controls. Furthermore, the authors found a main effect of the quantity of zeros within a number in the patient group, while this variable did not significantly affect the performance of healthy controls. The VLSM results demonstrated that patients committing overwriting errors had lesions associated with the right insula as well as parts of the frontal inferior and Rolandic operculum extending posteriorly towards the superior temporal gyrus. Furthermore, the right hemisphere was also prominently associated with other error types involving the addition and omission of zeros^20^.

The main weakness of the presented neuropsychological studies is that they all selected patients with specific lesion sides, or a specific behavioural problem in small sample groups or single cases. Therefore, their results can inform us about the phenomenon of zero errors but the limited power across different brain regions does not allow us to draw firm conclusions about the neuroanatomical origins. To this day and based on the knowledge of the authors, no study has yet made the comparison of errors in transcoding numerals in a large unselected and unbiased (not based on behaviour or lesion locations), sample of patients.

The present study aimed to take a large sample of patients with various lesions in the left and/or the right hemisphere, without pre-dividing them into lesion groups or selecting them based on behavioural characteristics, and to find out which lesions correlate with errors in transcoding zeros from a verbal dictation input to a written digit output level. We aimed to rigorously test this by using an unbiased VLSM approach, known to yield valuable insights into the relationships between brain tissue damage and behaviour on a voxel-by-voxel basis^21^. In this analysis, we compared patients with no transcoding problems (neither reading nor writing impairments) with patients who selectively show zero transcoding errors but do not commit any other errors in writing numerals.

## Results

### Behavioural results

The descriptive analysis of the behavioural data showed that 45.1% of the tested subacute stroke patients did not show any impairment in reading or writing numerals. 28.8% of patients showed combined reading and writing impairments. A few patients (8.3%) presented with a reading but no writing impairment. The errors committed by the patients who had impairments in writing but a perfect reading performance (17.8%) were analyzed further. In the number writing task, 14.2% of the patients showed a pure zero error in number transcoding while 16.0% committed one or more other number transcoding errors but no zero errors. These other errors consisted of swapped digits, perseverations (others than “0”), wrong spellings, missing parts, inserted or added digits (not “0”) as well as writing down one or more wrong digits. 6.7% of the patients showed impairments in zero transcoding as well as at least one other error category (see Table 1).

**Table 1:** Behavioural data of all patients (n = 667).

	Number of Patients
No writing or reading impairment	301
Both writing and reading impairment	192
No writing but reading impairment	55
No reading but writing impairment	119
Zero error but no other writing errors	95
Zero error and one other writing error (sum, consisting of:)	45
perseveration of digits other than zero	5
spelling error	6
random digits inserted or added	2
one digit wrong	10
more than one digit wrong	4
missing parts of one or more digits	16
swapped digits	2
mirrored digits	0
random letters inserted or added	0
No zero error but other writing errors (not additive because several error types are possible per patient)	107
perseveration of digits other than zero	19
spelling error	10
random digits inserted or added	13
one digit wrong	30
more than one digit wrong	19
missing parts of one or more digits	21
swapped digits	5
mirrored digits	3
random letters inserted or added	2

For the main lesion analysis of the present study, a sub-sample of 153 patients was analysed (see inclusion criteria). 106 (69%) of those patients did not show any writing or reading impairments and were considered the control group, while 47 patients (31%) committed selective zero transcoding errors. Both groups differed significantly in their age (*U* = 1944.000, *z* = −2.164, p = 0.030, *r* = −0.175) and years of education (*U* = 1957.000, *z* = −2.138, p = 0.033, *r* = −0.173), with the patients who made zero errors having a higher age and lower education level. For this reason, age and years of education were added to the VLSM analysis as covariates. No significant group differences were found for the variable sex (χ^2^(1) = 1.077, p = 0.299), though there was a significant group difference for the variable handedness (χ^2^(2) = 6.973, p = 0.031) resulting from three self-reported ambidextrous subjects in the patient group committing zero errors while no ambidexterity was reported in the control group. Given known neuroanatomical differences for different sex and handedness^22, 23^ both were also added as covariates.

**Figure 1:**
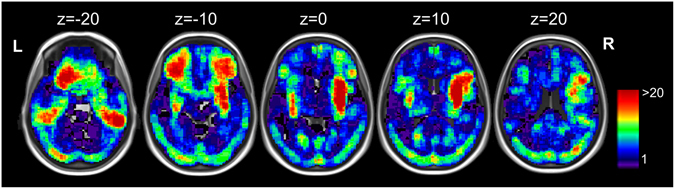
Lesion overlap of all patients (*n* = 153) presented on axial slices from caudal (z = −20) to cranial (z = 20) from a standard MRI template. Scale bars represent t-scores. L: left hemisphere, R: right hemisphere. Note that z-coordinates relate to MNI space.

Lesion overlay maps show the lesion distribution of the sample included in the VLSM, demonstrating adequate coverage and power and the inclusion of lesions in the left as well as right hemisphere (see Fig. 1).

The results of an additional exploratory analysis with regards to generalised differences in attention impairments between patients, grouped based on their transcoding errors, demonstrated that patients committing the specific zero transcoding errors had a significantly decreased mean accuracy score in the auditory attention task compared to patients without any reading or writing impairments (*U* = 1262.500, *z* = −4.692, p < 0.001). However, in comparison to patients committing perseveration or insertion errors with digits other than zero, they performed significantly better on this selective and sustained attention task (*U* = 201.000, *z* = −2.199, p = 0.028).

### Voxel-based lesion symptom mapping results

**Figure 2:**
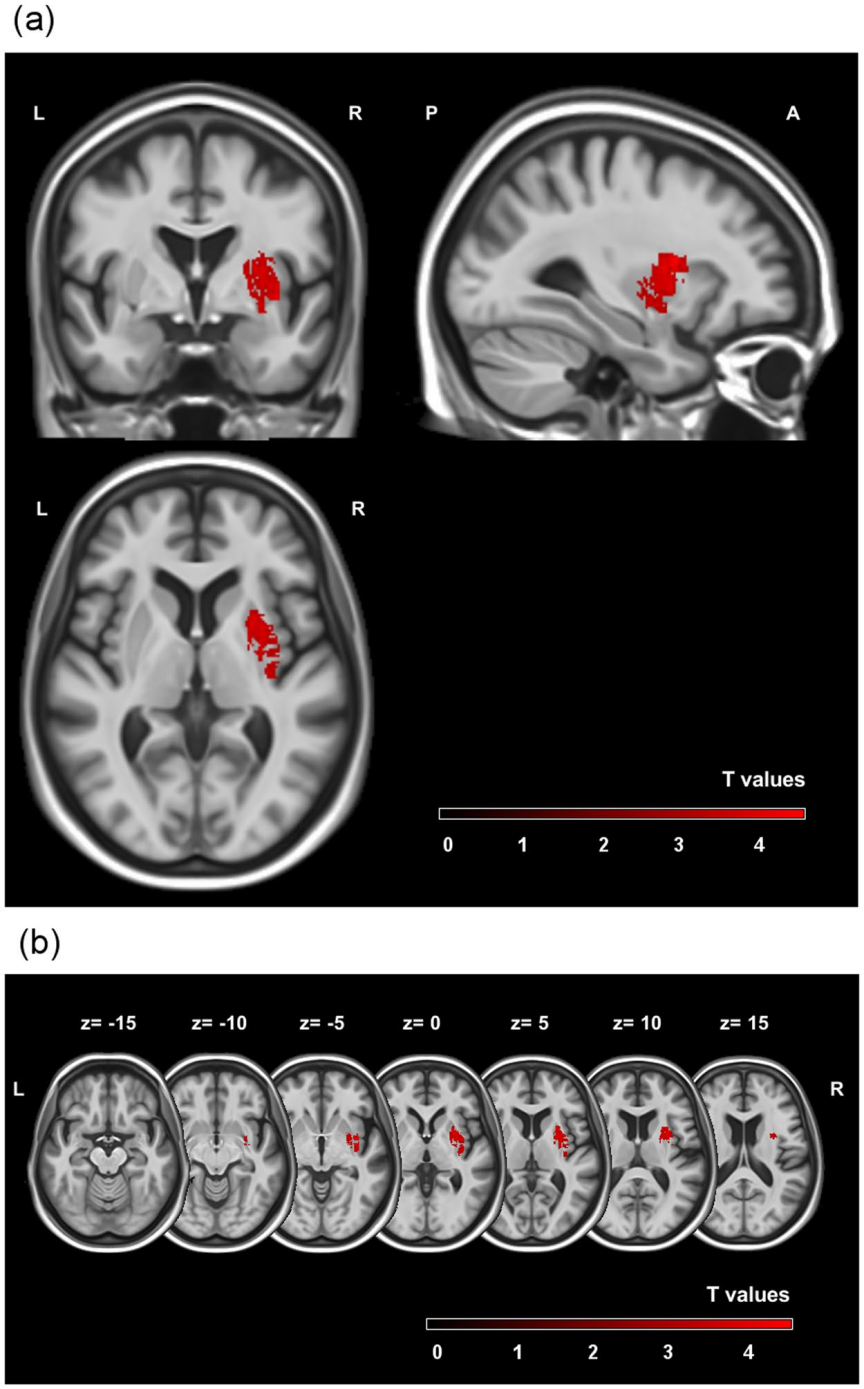
Significant cluster (**1**) of the voxel-based lesion symptom mapping analysis presented in an overview map (**a**) and on axial slices from caudal (z = −15) to cranial (z = 15) from a standard MRI template (**b**). The maps show permutation-corrected results (*p* < 0.05) at cluster level and with a threshold of *p* < 0.001, overlaid on a standard MRI template. Scale bars represent t-scores. L: left hemisphere, R: right hemisphere, A: anterior, P: posterior. Note that z-coordinates relate to MNI space.

**Figure 3:**
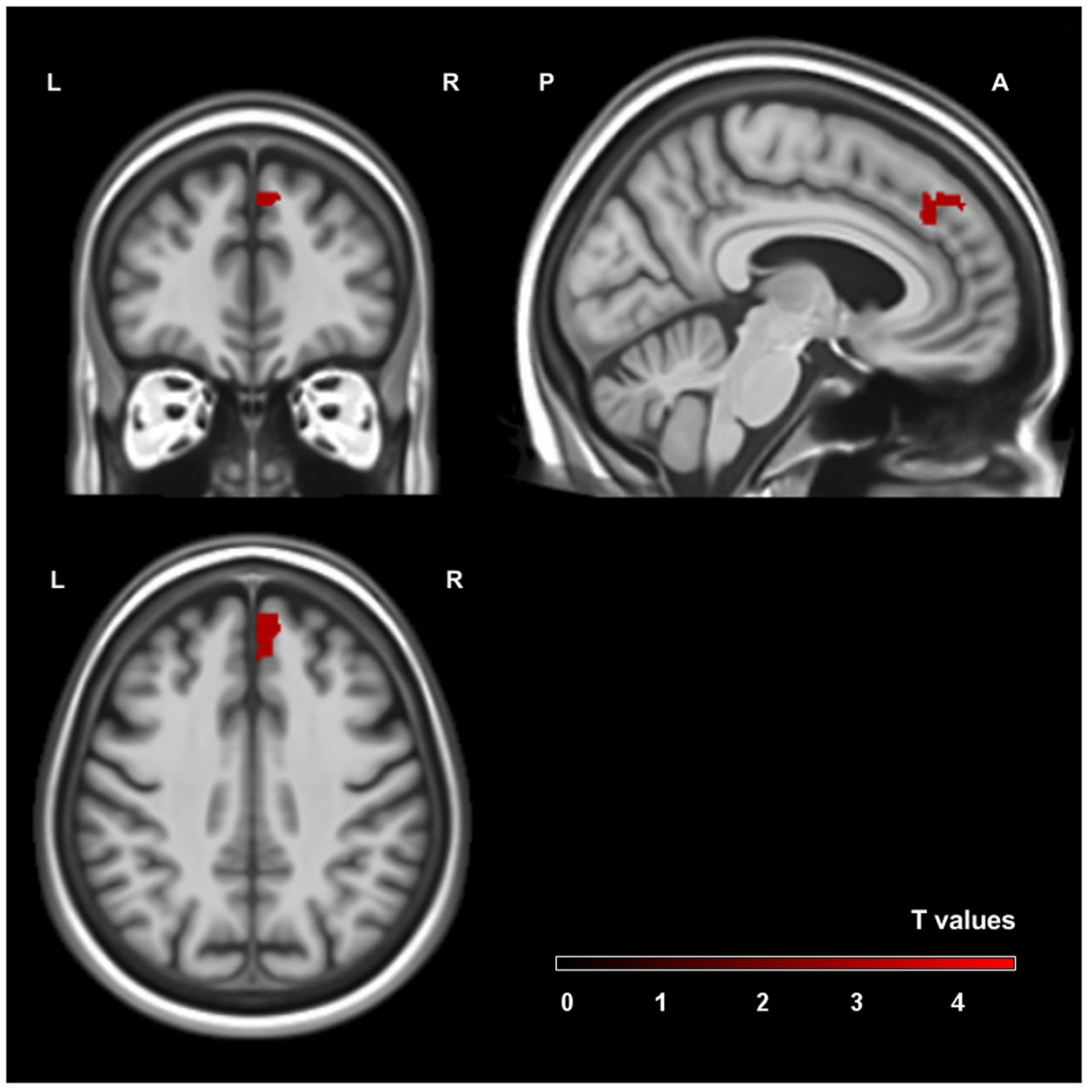
Second cluster (**2**) of the voxel-based lesion symptom mapping analysis. The area is significant at cluster level (*p* < 0.05) without permutation-correction with a voxel threshold of *p* < 0.001. Scale bars represent t-scores. L: left hemisphere, R: right hemisphere, A: anterior, P: posterior.

The main lesion analysis revealed a single significant cluster in the right putamen extending to the right insula (see Table 2 and Fig. 2). The second biggest cluster was found in the anterior division of the cingulate cortex and is displayed for a complete overview of the results, though it did not reach significance after permutation-correction (see Table 2 and Fig. 3).

**Table 2:** Locations of the two biggest clusters.

Region	Hemisphere	MNI coordinates			*t*	Cluster size	*P*_Permutation_value cluster
		x	y	z			
Putamen (1)	right	31	−1	3	4.67	3914	0.022
Anterior Cingulate Gyrus (2)	right	5	39	37	3.20	738	0.375

Note. Voxel coordinates are in millimeters after conversion to Montreal Neurological Institute (MNI) stereotactic space. On the voxel level, a high threshold of *p* < 0.001 was used for the analysis. All results are permutation corrected for multiple comparisons (*p* < 0.05).

**Figure 4:**
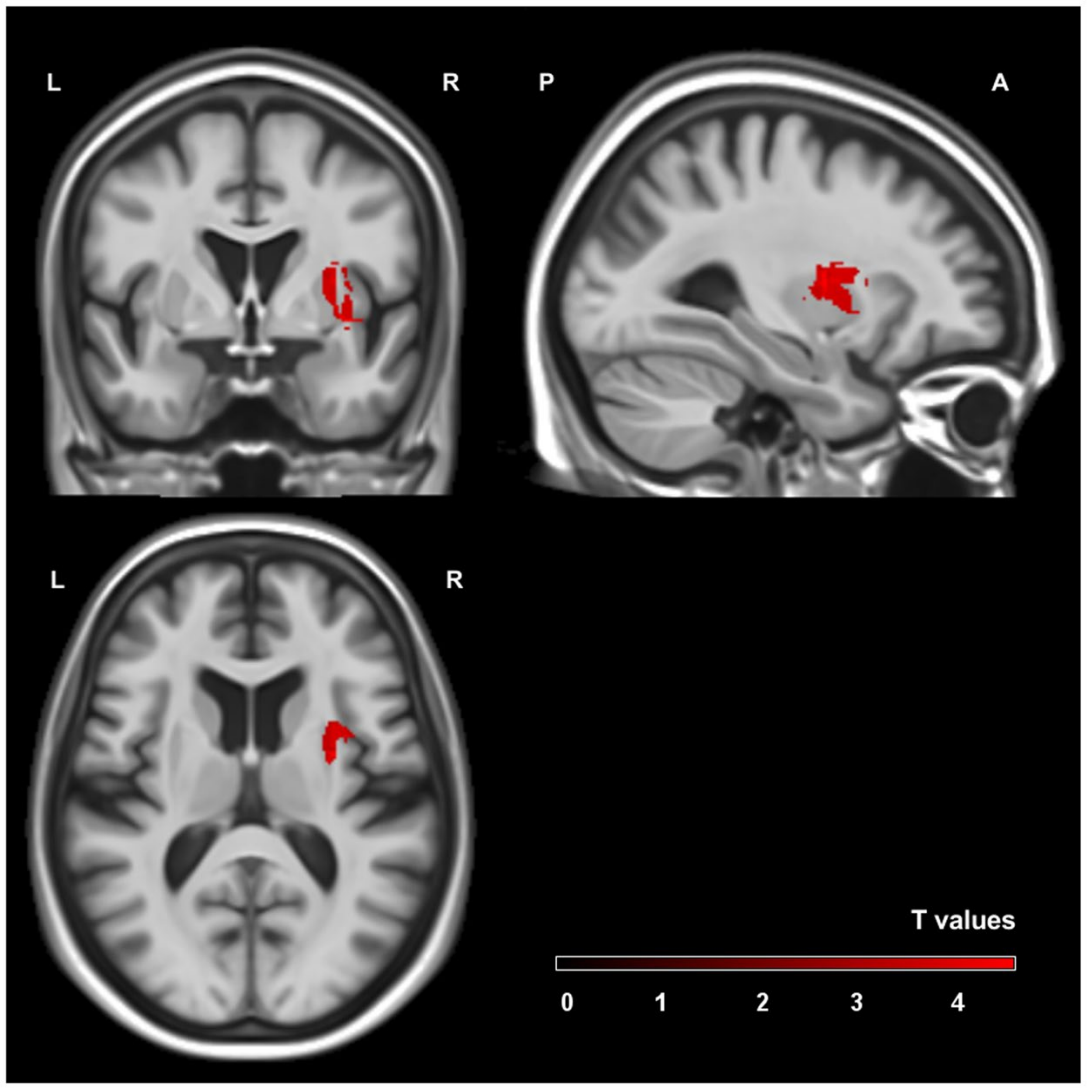
Cluster of the exploratory voxel-based lesion symptom mapping analysis. The area is significant with a voxel threshold of *p* < 0.01. Scale bars represent t-scores. L: left hemisphere, R: right hemisphere, A: anterior, P: posterior.

The additional analysis comparing the group of patients with zero transcoding errors (n = 47) to patients committing perseveration or insertion errors with digits other than zero (n = 16) did not reveal significant clusters after permutation correction. Nevertheless, a cluster comparable to the one found in the main analysis can be seen on an uncorrected level (p = 0.01; see Fig. 4).

## Discussion

The present study set out to investigate neuroanatomical lesions correlating with errors in transcoding zeros from oral dictation to written digits.

The behavioural results of the present study showed that errors in transcoding zeros are a lot more common than other transcoding errors. Overall, the percentage of patients solely making errors while transcoding zeros (14.2%) was nearly as high as the cumulative percentage of patients committing all the other possible errors (16.0%) while transcoding numerals. This result is in line with wide-spread models of numerical processing stating that out of all numerals, zero represents a special case because it is not represented on the semantic level^5^. Therefore, it might be prone to more transcoding errors than other numerals which have a semantic representation.

The results of the voxel-based lesion symptom mapping analysis were in accordance with our assumptions and in line with the study by Benavides-Varela *et al*.^20^. The significant cluster was localised in the right putamen extending to the right insular cortex. We did not find a significant parietal or other left hemispheric lesion to be the cause of the zero transcoding errors. This result cannot be explained by a limited sample only including patients with right hemispheric lesions, as was demonstrated in Fig. 1, the sample’s lesion distribution was spread over both hemispheres.

We interpreted this finding in line with a PET study showing that the putamen was activated in counting tasks with and without auditory stimuli^24^. This study indicated that the putamen is not just involved in tasks with auditory stimuli but, beyond that, plays a role in processes underlying counting. The authors interpreted the significantly right lateralized putamen activity during non-auditory counting as an indication of this region being involved in sustained attention tasks requiring working memory^24^. Right hemisphere dominance during attention as well as specific auditory attention tasks has been suggested repeatedly in literature^25, 26^. More support for this interpretation comes from a PET study comparing the processes of calculation and mere number repetition^27^. During calculation, activation in the medial frontal/cingulate gyri, left dorsolateral prefrontal cortex, left anterior insular cortex and right anterior insular cortex/putamen, left lateral parietal cortex, and the medial thalamus was found. In contrast to that, number repetition, lower in attentional and working memory load, resulted in bilateral inferior sensorimotor cortex, bilateral temporal areas, and left inferior frontal cortex activations. The specific activation patterns for calculation suggest a functional anatomical network including various aspects: attention, working memory components (phonological store and articulatory loop) as well as auditory and motor processing^27^. With this, the study supports the established knowledge of the parietal cortex’ special role and the existence of the involved extensive parieto-fronto-cingular network in calculation tasks^28^. When we considered our VLSM results without permutation correction to explore the data further, we found a cluster in the anterior cingulate gyrus which supports the role of this network. Therefore, we assume that patients have a comparable attention and working memory load in transcoding zeros and other numerical processing tasks like counting or calculating. Besides, the parieto-fronto-cingular network might play an especially important role for committing errors in transcoding zeros because the prefrontal cortex is suggested to be the key area for a concept of zero and the integration of its numerosity as the lower end of our numerical continuum^29^. Hence, it seems like both, the concept of zero numerosity and the necessary attentional resources for transcoding numerals including zeros, have to be integrated in the same network to enable a successful performance. Apart from that, the results underline that subcortical areas, like the putamen, play an important role in this network.

The extension of the significant cluster to the insular cortex is also in line with results of a former VLSM study on zero errors in number transcoding by Benavides-Varela *et al*.^20^. The authors interpreted the result as the insula’s integrative role in a wide range of cognitive functions being well aligned with the overwriting rule as part of the lexical-semantical model proposed by Power and Dal Martello^3, 4^. Furthermore, this result from transcoding studies is congruent with an activation found in the same area while subjects worked on addition tasks^13^. The insula’s activation is thought to be connected to memory-based fact retrieval mechanisms. At the same time, this region is indispensable for auditory perception and the sensory integration of this auditory information^30^. In favour of this theory, a significant activation of the insula was found when subjects were asked to remember written letters by transforming them in phonological code and sub-vocally rehearsing them^31^. This account does not just hold for letters but also numbers. When asked for transcoding digits to orthographically written numbers, patients with parietal lesions did not make any errors while they showed impairments in other processing domains^32^. Moreover, the same study could show right hemisphere dominance in processing digits^32^.

Another reasonable explanation for the association between the circumscribed lesion and an increased number in zero errors can be found with regard to acetylcholine. The lesion location includes the external capsula which is known to contain fibers carrying acetylcholine to the cortex. Apart from playing a vital role in learning and short-term memory functions, acetylcholine is essential for top-down control of attentional orienting and the discrimination of stimuli^33^. Future studies are needed to shed light on the relation between attentional top-down control parameters and zero errors.

Nevertheless, patients exclusively committing zero errors did not show more generalised attentional deficits (as measured in an independent auditory selective attention task) compared to patients committing insertion and perseveration errors with other digits. In addition, the exploratory VLSM analysis shows that solely zero errors are significantly associated with lesions in the right putamen. Hence, general attention resources are unlikely to be the only intermediary cognitive function of the lesion and the behavioural symptom. This points in the direction of an interplay of higher cognitive functions being required to perform this specific number transcoding command which cannot solely rely on semantic input.

Interestingly, the capsula externa is also associated with white matter fiber tracts which belong to the fronto-occipital fasciculus. The left inferior stream of this tract subserves language semantics^34^. In addition, left-hemispheric white matter tracts connecting frontal, parietal, and temporal regions such as the superior longitudinal fasciculus were found to be associated with individual differences in learning mathematical abilities^35^. White matter tractography may represent a promising tool in further examining the underlying processes in transcoding numbers.

We note that the number of days between the stroke and the acquisition of the CT scan is a limiting factor in regard to the actual lesion size. The CT scans in the present study show the core of the lesion but, due to diaschisis, may fail to identify the actual size^36^. Future studies should control for these effects by acquiring an additional brain scan at the time of behavioural testing. Nevertheless, the replication of the results by Benavides-Varela *et al*.^20^ adds weight to the indication of a decisive role of the right putamen and insular region.

Furthermore, we remark that the discussed neuroanatomical regions share a similar vasculature, being mainly supplied by the middle cerebral artery. Even if this holds potential for detecting a specific location because it is the root that supplies damage to a distributed network^37^, we do not assume that this is the underlying explanation for our results. Firstly, the VLSM results do not only cover posterior but also anterior parts of the putamen which get additional blood supply by the anterior cerebral artery. Secondly, Benavides-Varela *et al*.^20^ find comparable VLSM results which, however, are solely located in the right insula. Hence, the analyses do not seem to be prone to yield vast lesion locations which are remapped based on a common vascular root.

In sum, the parieto-fronto-cingular network appears to be essential for number processing with lesions in, especially the left, parietal lobes leading to severe impairments. Not just cortical but also subcortical areas seem to play a crucial role for cognitive processes underlying number processing, though. In accordance with former research, the present study underlines that the right putamen as well as the right insula are important components of attention, working memory and auditory processes. Based on our results and in line with the finding from Benavides-Varela *et al*.^20^, a lesion in these right hemispheric areas led to errors in transcoding zeros in numerals.

However, this finding does not mean that impairments in transcoding zeros have to be permanent. A single case study showed that a patient’s error percentage in transcoding syntactic zeros, decreased from 54.2% in the first session (8 weeks post stroke) to 6.5% in the fourth session, 84 weeks after his stroke^5^. All the patients in the present study were assessed in one testing session in their sub-acute phase. Therefore, future investigations could focus on examining patients in various sessions over the time course of their rehabilitation process to find out whether impairments in transcoding zeros do, indeed, decrease.

Future research should extend these findings by contrasting different types of transcoding errors in a large, and unbiased patient sample to understand if the underlying lesion is specific to errors in transcoding zeros due to the missing semantical representation and possibly higher attentional load. In addition, broader questions need to be asked about the mechanisms underlying and mediating the relation between number transcoding and broader top-down attentional processes as well as the role of white matter tracts.

In conclusion, the present study, for the first time, linked neuroanatomical lesions in the putamen and insula to behavioural impairments in transcoding zeros in a large unbiased sample of sub-acute stroke patients, providing the best powered and strongest methodological evidence available to date. Bearing in mind that zero forms a complex mathematical concept rather than solely being a number, it is plausible that it takes up a special role in the process of number transcoding. Due to the mere fact of a missing semantical representation of zero, an additional transcoding rule, namely overwriting, is needed. The late evolvement of mastering this rule in development as well as its performance decrease in neuropsychological patient populations point into the direction of it demanding higher cognitive efforts. The results of the present study underline this notion by showing that the main structure associated with zero transcoding errors is the right putamen with an extension to the right insula. The cognitive mechanisms behind these areas as well as their connections to a bigger parieto-frontal network are mainly based on merging and integrating different sources of information as needed for tasks with a higher cognitive load and attentional demand. Therefore, the present neuropsychological lesion to function mapping findings enabled a better understanding of the necessary neuro-anatomical processes that support the way zero is being transcoded.

## Materials and Methods

### Participants

The recruited patients were part of the Birmingham Cognitive Screening project and completed the BCoS^38^ within 3 months post stroke. Several stroke units across the West Midlands area of the UK participated in this large clinical study. Patients with missing behavioural data on the reading and writing tasks were excluded for the behavioural analysis. Our final sample for the behavioural analysis consisted of 667 subacute stroke patients (see Table 3). Their clinical and demographic data was obtained from their medical notes. For the VLSM analyses, the inclusion criteria were: (i) an available CT scan with a clear circumscribed lesion present, and (ii) no general impairments in writing numerals (e.g. formation of numbers or illegible writing) (see Table 3). This resulted in 153 patients that were included in the main VLSM analysis.

**Table 3:** Patient details: Clinical and Demographic Data.

	Mean Value or Number of Patients	*SD*	Range
BCoS Behavioural Sample (*N* = 667)			
Age in years	69.2	13.9	18.0–94.0
Sex (male/female)	379/288		
Handedness (right/left/ambidextrous)*	584/66/14		
Education in years**	11.5	2.7	3.0–25.0
VLSM Sample (*n* = 153)			
Age in years	70.1	13.3	27.0–92.0
Sex (male/female)	78/75	—	—
Handedness (right/left/ambidextrous)	141/9/3	—	—
Education in years	11.9	2.9	5.0–24.0
Time Stroke-BCoS in days^a^	24.2	21.9	1.0–91.0
Time Stroke-CT in days^b^	1.9	3.0	0–20.0
Lesion side (right/left/bilateral)	84/43/26	—	—
Etiology (ISCH/BL/O)^c^	126/22/5	—	—

Note. VLSM: voxel-based lesion symptom mapping; BCoS: Birmingham Cognitive Screen; ^a^time between stroke and assessment of the BCoS in days; ^b^time between stroke and computer tomography in days for n = 131; ^c^ISCH = ischemic stroke, BL = bleed (haemorrhagic stroke), O = others; *missing values for 2 patients; **missing values for 6 patients.

Additional analyses were conducted comparing the patients committing solely zero errors (n = 47) with patients committing perseveration or insertion errors with digits other than zero (n = 16).

The CT scans were taken as part of the routine clinical assessment following a stroke and the admission to the hospital. The National NHS ethic committee and local NHS trusts approved the experiment. All methods were performed in accordance with the ethics protocol’s relevant guidelines and regulations. Written informed consent was obtained from all study participants in agreement with the ethics protocols.

### Behavioural Measures

#### Cognitive Profile

The neuropsychological testing of patients took place in a hospital setting during the subacute phase following stroke onset (<3 months post stroke) with the average stroke to test interval being 24.2 days (*SD* = 21.9). The BCoS cognitive profile of each participant is composed of five broad cognitive domains^39^. The assessment lasted around one hour, and comprised of 23 tests. In the present study we focused on two of the number abilities domain subtests which assessed the number reading and writing abilities of patients. Additionally, the accuracy in the Auditory Attention subtask of the BCoS was included in a exploratory analysis to investigate more general attentional differences between the different patient groups. In this task, participants are required to make selective tapping responses to target words (hello, please, no), whilst withholding responses to non-target words (goodbye, thanks, yes) over a period of three minutes^38^.

#### Number writing and reading

The task in focus for this manuscript is the number writing task, which was comprised of five items (807; 12,500; £5.99; £25.50, £329.89). These numerals consisted of units of hundreds and thousands, additive and multiplicative relations, as well as embedded zeros. Numbers were read out loud to the participant who was requested to write them down as indicated (in digits, not spelled out). The number was systematically repeated once whilst the participant was writing to not load memory.

The number reading task included a total of nine items which were divided in three categories: complex numbers, prices, and times. These numbers were presented in sets of three large font letters in the centre of a page to limit potential confounds of spatial unilateral neglect and participants were simply instructed to read aloud the numbers on the page. The examiner noted their responses. The three complex numbers were 539; 2,304; and 17,290. Functional measures of the processing of numbers in everyday situations were assessed by reading out three prices (£3.99; £109.50; £724.89) and three times (9:30; 2:45; 6:10).

### Image Pre-processing

CT image pre-processing of all patient data was performed using SPM8 (the Wellcome Trust Centre for Neuroimaging, London, UK) and an automated lesion delineation software written in MATLAB (The Math-Works, Natick, MA, USA). The lesion delineation software applied a threshold-based clustering at 0.1% maximum intensity before spatially aligning the resulting CT image to template image via the SPM8 co-registration tool^40^. In a next step, the CT image intensity was transformed. The transformed CT images were warped to MNI space. Then, the normalised image was resliced at 1mm isotropic resolution including the cortex as well as the cerebellum. In SPM8, this normalised image was smoothed using a Gaussian filter. This final pre-processing step aims to accommodate the assumption of random field theory in statistical analyses^40, 41^.

### Voxel-based lesion symptom mapping

The voxel-based lesion symptom mapping analysis was performed using the software of Bates *et al*.^21^ implemented in MATLAB 2012a (The Math-Works, Natick, MA, USA). Voxels in which fewer than five patients had a lesion were excluded from the analysis and lesion volume was automatically entered in the analysis as a covariate of no interest. T-tests were used to perform statistical comparisons on a voxel-wise basis using the performance measure (zero transcoding error versus no errors) as the dependent variable. The tests were run using permutation derived correction (with 3000 permutations) which is an assumption-free procedure and more powerful than others, such as Bonferroni correction^42^. For the present study, a voxel-wise threshold of p < 0.001 was used and the significance level for the corrected values was set to p < 0.05.

VLSM lesion peaks’ localisations were determined in MNI space using the Harvard-Oxford cortical and subcortical structural atlases (http://www.cma.mgh.harvard.edu). Visualisations were made using MRICron software (http://www.mccauslandcenter.sc.edu/mricro/mricron/index.html)^43^.
